# The changing epidemiology of pediatric aseptic meningitis in Daejeon, Korea from 1987 to 2003

**DOI:** 10.1186/1471-2334-5-97

**Published:** 2005-11-02

**Authors:** Kyung-Yil Lee, David Burgner, Hyung-Shin Lee, Ja-Hyun Hong, Mi-Hee Lee, Jin-Han Kang, Byung-Churl Lee

**Affiliations:** 1 Department of Pediatric, College of Medicine, The Catholic University of Korea, Seoul, Korea; 2 School of Paediatrics and Child Health, University of Western Australia, Perth, Australia

## Abstract

**Background:**

Aseptic meningitis is a relatively frequent childhood disease and virologic data suggest that enteroviruses are the commonest etiologic agents. We evaluated the epidemiologic characteristics of aseptic meningitis in Daejeon, South Korea from 1987 to 2003.

**Methods:**

2201 medical records of children with aseptic meningitis admitted to The Catholic University of Korea, Daejeon St Mary's Hospital were retrospectively analyzed.

**Results:**

Outbreaks of aseptic meningitis were observed in 1990, 1993, 1996, 1997, 2001 and 2002. The age distribution of cases was relatively uniform, with a higher incidence in those aged < 1 year and 4–7 years. The male-to-female ratio was 2:1. There was a higher incidence of disease in the summer (May to August, 74.1% of total). Comparison of the largest epidemics in 1997 and 2002 showed significant differences in the incidence in those < 1 year (11.8% vs. 4.4%, respectively; *P *= 0.001). Neurologic sequelae were observed in 0.7% of the patients.

**Conclusion:**

Aseptic meningitis, rare before the 1980s in Korea, has since become a common clinical entity. Since 1990, outbreaks of aseptic meningitis have occurred every 1 to 3 years in Daejeon in keeping with Korea-wide epidemics. The frequency of disease affecting children less than one year of age may reflect herd immunity to the epidemic strain.

## Background

Aseptic meningitis is a relatively common disease of childhood and recent data suggest that it is most frequently caused by enteroviruses [1,2]. Although globally aseptic meningitis occurs year-round, there is a marked seasonality, with an incidence peak in the summer months in temperate climates. In Korea, epidemics of aseptic meningitis have occurred approximately every 3 years since the early 1990s. Whilst there have been a number of studies addressing the clinical characteristics and the causative agents of these epidemics [3-15], long term epidemiologic studies of aseptic meningitis are rare. A variety of enteroviruses have been suggested as the predominant causative agents for each Korean epidemic, with enterovirus 71 or echovirus 30 in 1990 [6], echovirus 9 in 1993 [7], echovirus 30 in 1997 [11], echovirus 6 in 1998 [11], coxsackievirus B5 in 2001 and echovirus 13 in 2002 [12-14].

In this study, we describe the epidemiologic characteristics of pediatric aseptic meningitis during a 17 year period, from 1987 to 2003, in Daejeon, South Korea, with particular emphasis on the two largest epidemics, in 1997 and 2002.

## Methods

We retrospectively analyzed a total of 2201 medical records of pediatric patients with aseptic meningitis patients admitted to The Catholic University of Korea, Daejeon St. Mary's Hospital from January 1987 to December 2003. Daejeon, located in central Korea, is one of the largest cities with a population of over 1.4 million. The majority of the patients with aseptic meningitis were admitted to one of four general hospitals in Daejeon. There were no significant changes of medical facilities or the social environment during the study period. Epidemic years were defined as those with more than 100 patients per year and at least a two-fold increase in cases compared to the previous year. The diagnosis of aseptic meningitis was made on the basis of: (i) clinical symptoms and signs of meningitis, such as fever, vomiting, headache, and meningeal irritation, (ii) cerebral spinal fluid (CSF) pleocytosis (≥ 5 leukocytes/mm^3^) with normal CSF protein and sugar levels and (iii) negative results on bacterial culture and latex particle agglutination test. Studies to identify viral pathogens were not routinely undertaken in these hospitals, especially during epidemics, as the available data suggests that causative agents other than enteroviruses are relatively rare [6,7,11-15]. Other non-enteroviral causes of meningitis occurring during the study period were excluded from the current analysis. These included cases of mumps meningitis (n = 66; 1989–1998) [16], bacterial meningitis (n = 40; 1992–2002) [17] and herpes meningo-encephalitis (n = 3). We analyzed the age and gender of patients, together with monthly and annual frequency. In addition, we evaluated the frequency of neurological complications among the patients hospitalized for more than 10 days. The epidemiologic features of the patients from the two years with the highest incidence, 1997 and 2002, were also compared.

## Results

### Age and sex distribution

**Figure 1:**
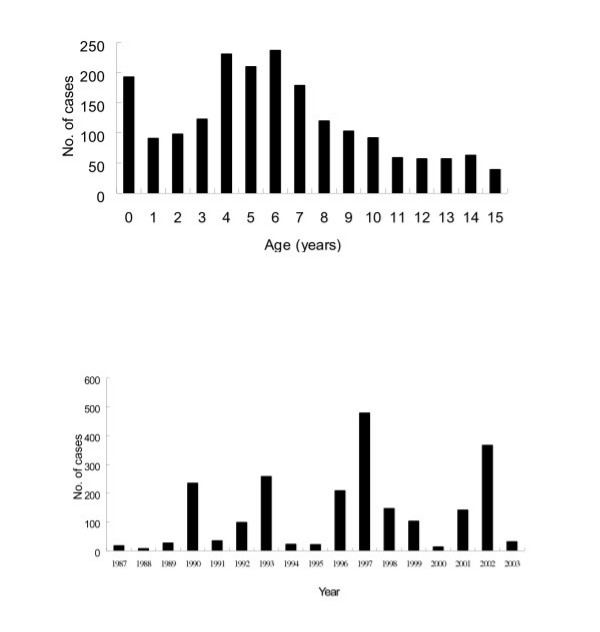
(A) Age distribution of aseptic meningitis, 1987–2003. (B) Annual cases of aseptic meningitis, 1987–2003.

2201 children fulfilled the study criteria. The mean age was 6.0 ± 3.9 years (range 2 weeks to 15 years). The highest incidence was in those aged 4 to 7 years (44.1% of total cases) and in those less than 1 year old (10% of total), with the remaining cases evenly distributed across the remaining ages (Fig. 1). There were 1470 males and 731 females, giving a male-to-female ratio of approximately 2:1. Although enteroviral studies were not performed routinely during the study period, enteroviruses were identified in a proportion of patients in the 1997 and 2002 epidemics (Table 1). The mean duration of hospitalization was 5.5 ± 1.7 days. There were no fatalities.

**Table 1:** Enteroviruses isolated from each nationwide epidemic of aseptic meningitis in Korea.

Year	Cases	Specimen(s)	Enteroviruses isolated (n)	References
1990	118	Serum	Suspected Enterovirus 71 & EV 30	6
1993	93	CSF	EV 9 (60), Others (17)	7
1996	210	Stool, CSF	EV 9 (11), CV B1 (6)	11
1997	493	Stool, CSF	EV 30 (54), EV 6 (15), CV B5 (9), Others (3)	11
1997	33	CSF	EV 30 (6)	*
1998	341	Stool, CSF	EV 6 (36), CV B2 (11), Others (29)	11
2002	371	CSF	EV 13 (18), EV 9 (15), EV 6 (10), Others (24)	13
2002	13	Stool, CSF	Phylogenetic analysis of EV 13	14
2002	29	CSF	EV 13 (5), EV 6 (1)	*

CSF, Cerebrospinal fluid; EV, Echovirus; CV, Coxsackivirus;

*, Unpublished data of our study

### Annual incidence

The number of aseptic meningitis cases per year ranged from 17 (0.8% of total cases) in 1987 to 489 (21.7%) in 1997; the average was 129 cases per year. The greatest number of cases were observed in 1997, 2002 and 1993, with 489 (21.7%) of total, 366 (16.6%) and 257 (11.7%) patients, respectively. Outbreaks occurred approximately every three years in 1990, 1993, 1996, 1997, 2001 and 2002 (Fig. 1).

### Monthly and seasonal frequencies

**Figure 2:**
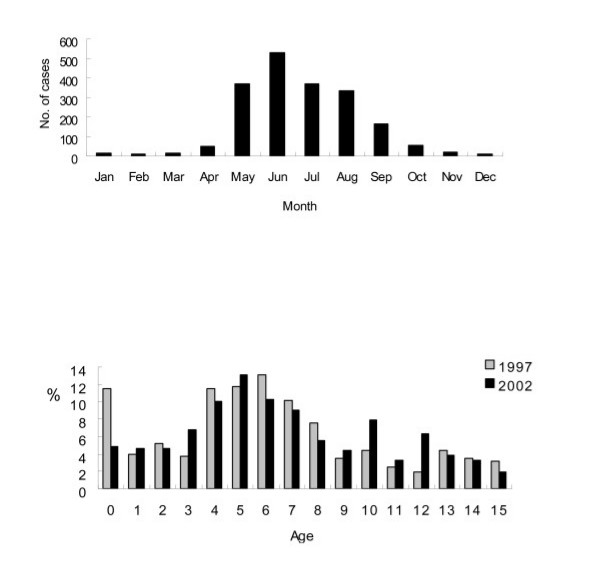
(A) Monthly cases of aseptic meningitis, 1987–2003. (B) Comparison of age distribution between in 1997 and in 2002.

There was a striking seasonal pattern, with almost three quarters of cases occurring during the summer months (May to August) (Fig. 2). Of the total of 2201 cases, 540 (24.5%) presented in June, 378 (17.2%) in July, 371 (16.9%) in May, and 341 (15.5%) in August (Fig. 2).

### Comparison of epidemiologic features of the epidemics in 1997 and 2002

1997 and 2002 had the largest epidemics of aseptic meningitis in the study period. There was no significant difference between in these two large epidemics in the age of children and the monthly distribution. However, in 1997, there was a higher frequency in those < 1 year of age than in 2002 (11.8% vs. 4.4%, *P = *0.001, *X*^2 ^test) (Fig. 2).

### Neurologic sequelae

Medical records of the 127 children hospitalized for ≥ 10 days were analyzed for possible complications. Although other complications of the gastrointestinal or respiratory system were noted, only neurological complications were evaluated in this study. Neurological complications were noted in 16 patients (0.7% of total). All children with neurological complications had abnormal mental status or abnormal neurological symptoms and signs following their aseptic meningitis and had been neurologically normal previously. Seizures were observed in 5 children, of whom two developed status epilepticus. Transient amnesia was observed in 2 children; one had antegrade amnesia, and the other had retrograde amnesia. One child had an inappropriate secretion of anti-diuretic hormone and mild hydrocephalus. One child showed transient signs of quadriplegia. Thirteen of the 16 children with neurological complications were followed up for at least 2 months. No permanent neurological sequelae were observed.

## Discussion

Although there have been a number of studies of aseptic meningitis in Korea [3-15], there are few epidemiologic studies of this scope and duration. Historically, aseptic meningitis appears to have been relatively unusual in Korea prior to the 1980s, but this may partly reflect ascertainment bias and diagnostic methods. Chung et al. described 104 cases of childhood meningitis from 1960–1967, with 41 cases of aseptic meningitis cases and 63 bacterial meningitis cases [3]. Rheu et al. reported an increasing incidence of aseptic meningitis in the Wonju area of Korea from 1966 to 1983; among the total of 200 cases of aseptic meningitis, there were 1–3 cases per year from 1966–1971, 6–23 cases per year in 1972–1982, and 40 cases in 1983 [4]. Similar findings are reported from Busan during the 1980s, with 5–19 cases annually between 1980 and 1988, 30 cases in 1989 and 56 cases in 1990 [5]. These results suggest that the nationwide epidemics of aseptic meningitis in Korea started in the early 1990s. The striking change in epidemiology at this time, with the occurrence of epidemics, may partly reflect improved diagnosis and increased awareness. In addition, changes in herd immunity may have contributed to an increased population susceptibility to epidemics of virus transmitted by the fecal-oral route. Public health improvements that accompanied this period of marked economic growth in Korea may have reduced sporadic exposure to enteroviruses and led to a large non-immune population. Similar patterns have been observed in the rapidly declining seroprevalence of hepatitis A in Korea. Hepatitis A, which is a kind of enteroviruses and also spread by the fecal-oral route, currently has a seroprevalence in Korean children < 15 years old of almost zero [18,19].

In this study, epidemics of aseptic meningitis in Daejeon mirrored the nationwide epidemics in 1990 [5,6], 1993 [7,8], 1996 [9], 1997 [10], 2001 and 2002 [12-14]. A nationwide study of 5090 patients in 1993 showed that the epidemic of aseptic meningitis commenced in the southern regions in spring, and then gradually moved to the northern regions (including Seoul), reaching a peak incidence in summer and then waned by late fall [8]. Similar patterns have been observed in other epidemics in Korea [5,6] and may reflect the geographical and social environment with a large and mobile population contributing to the nationwide spread of the disease. Aseptic meningitis is generally commoner in males with a male-to-female ratio of 1.2–2.3 to 1 [4-13], as observed in the current study.

Globally the age distribution during epidemics of aseptic meningitis varies, possibly due to different causative agents and specific herd immunity that results from varying socio-economic environments and other factors. For example, in the United States, the peak age for children with aseptic meningitis is reported to be < 1 year old [1,2,20], whereas in five South African epidemics in the 1980s, the mean age of children with echovirus meningitis was 4–5 years of age, whereas those with coxsackie B meningitis were most commonly <1 year old [21]. In Japan, Yamashita et al. analyzed 8595 cases of aseptic meningitis from 1981–1991. There were two peak ages of < 1 year and 4–7 years, with varying age distributions according to the causative virus in each epidemic [22]. Although Korea and Japan have similar geographical, racial and socio-economic environments, the period of marked Korean economic growth occurred later than in Japan. The Korean epidemiologic data from the 1990s is similar to those from the 1980s in Japan [22,23], possibly reflecting similar changes in important determinants of  epidemiology.

The pattern of age distribution in an aseptic meningitis epidemic may reflect herd immunity from past infection with the same causative virus. Aseptic meningitis is rare in younger adults in Korea, whereas adults aged 20 and 40 years have an incidence similar to children in the United States [20]. In addition, there were differences in incidence in infants (< 1 year old) with each epidemic, suggesting that herd immunity from previous infections with enteroviruses is reflected in protective transplacental antibodies. Maternal (transplacental) antibodies are detected in over half of the infants aged 6 months, and may persist for up to 12 months after birth. Thus a high incidence in both infants and adults suggests a new epidemic strain with low herd immunity.

Previous studies in Korea have reported that enteroviruses predominate in each nationwide epidemic (Table 1). Enteroviruses include more than 70 serotypes, but only a few serotypes typically cause aseptic meningitis in any given community and during any given year. This may be due to differences in the background rate of infection in a community (herd immunity), in host immunity and possibly in the viral strain's neurotropism. Recently, echovirus 13 meningitis epidemics have been reported in Europe in 2000 [24,25], in the United States in 2001 [26], and in Korea in 2002 [12-14]. The genotype of echovirus 13 isolated in Korea in 2002 is almost identical to that isolated in Japan and Germany [14]. Thus, aseptic meningitis epidemics from an enteroviral strain may spread globally.

Children with enteroviral meningitis generally recover without complications, but rarely the disease can cause neurologic sequelae [1,2,8]. The clinical manifestations and outcome may differ with the enterovirus serotype. For example, a more severe clinical course and worse outcome with enterovirus 71 infections have been reported both in Korea and in other countries [27,28]. In the current study, 16 children (0.7%) with aseptic meningitis had neurological complications; 2 children in 1990, 4 in 1993, 4 in 1997, and 1 child each in six other years. No single epidemic resulted in a significantly larger number of neurologic sequelae, although the denominator was small as the outcome was generally excellent.

## Conclusion

Aseptic meningitis appears to have been a rare disease in Korea during the 1960s and 1970s. Since 1990, outbreaks of aseptic meningitis have occurred every 1–3 years in Daejeon in keeping with nation-wide epidemics. The changing epidemiology may reflect improvements in public health, characteristics of the predominant etiologic agents and changes in herd immunity. In particular, the incidence of aseptic meningitis in children less than one year of age in each epidemic may be indicative of the agent-specific immunity of the parental generation.

## Competing interests

The author(s) declare that they have no competing interests.

## Authors' contributions

KYL designed the study and drafted the manuscript. JHH, MHL and JHK participated in the data collection and analysis, HSL analyzed the final data. BCL participated in the supervising the execution of the study. DB assisted with data interpretation and in drafting the manuscript. All authors read and approved the final version of the manuscript.

## Pre-publication history

The pre-publication history for this paper can be accessed here:


